# Frequency of silent carotid artery stenosis in diabetics and its associated factors: An analysis in tertiary care hospital

**DOI:** 10.12669/pjms.36.6.2306

**Published:** 2020

**Authors:** Faiza Sadaqat Ali, Nimrah Bader, Bader Faiyaz Zuberi, Sabiha Banu

**Affiliations:** 1 Faiza Sadaqat Ali, FCPS. Senior Registrar, Medical Unit-II, Dow Medical College, Karachi, Pakistan; 2 Nimrah Bader, MD. R-II, Oklahoma State University Medical Center, Oklahoma City, OK, USA; 3 Prof. Bader Faiyaz Zuberi, FCPS. Medical Unit-II, Dow Medical College, Karachi, Pakistan; 4 Sabiha Banu, FCPS. Endocrinology Fellow, Internal Medicine Department, Aga Khan University, Karachi, Pakistan

**Keywords:** Diabetes mellitus, Diabetic vascular disease, Silent carotid artery stenosis, Stroke Risk

## Abstract

**Objective:**

To estimate frequency of silent carotid artery stenosis and its associated factors in diabetic patients attending a tertiary care hospital.

**Methods:**

This cross-sectional study was conducted in tertiary care Civil Hospital, Karachi from March 2019 to September 2019,. A total of 166 patients with Diabetes Mellitus were included in this study. Brief history was taken for the duration of DM, treatment, and smoking habits. Carotid artery stenosis (CAS) wafrs measured by Doppler ultrasound of right and left common, internal, and external carotid arteries.

**Results:**

Frequency of silent carotid artery stenosis (CAS) in diabetic patients was observed in 28.92% (48/166) cases. The mean age ±SD of the patients was 54.8 ±7.96 years. 27 (22.29%) patients were smoker and all were male. Out of 166 diabetic patients, 59 (35.54%) were treated with insulin and 107 (64.46%) were treated with oral hypoglycemic.

**Conclusion:**

Substantial number of diabetic patients with increasing age, increased duration of diabetes and smoking habits have significant silent Silent Carotid Artery Stenosis (CAS).

## INTRODUCTION

Stroke is an important reason of mortality in developed countries. Atherosclerotic stenosis of carotid arteries is one of the major contributing factors for ischemic stroke. Stroke contributes a high socioeconomic burden, it is ranked among the top three causes of morbidity and mortality globally.^1^ About 8-15% of ischemic strokes are associated with more than 50% stenosis of extra cranial internal carotid arteries.^2^ Burden of carotid artery stenosis (CAS) among diabetic patient is rising. The risk of cardiovascular diseases (CVD) including coronary and carotid artery disorders and stroke in diabetic patients is three times higher than in in non-diabetics.^3^ Risk of atherosclerotic disease has decreased in recent past due to better medical management, however patients with severe stenosis of carotid artery still have a relatively high risk of complications.^4^-^6^

Cardiovascular diseases (CVDs), including stroke and ischemic heart disease (IHD), are now the prime reason of early sudden death in Western and Chinese populations.^7^ Since atherosclerosis progress preclinically over a period of decades prior to clinically evident CVD, so population based surveys utilizing carotid artery ultrasonography, highlighting carotid artery disease may give useful information about future risks of CVD.^8^ Several studies have demonstrated the presence of carotid stenosis in diabetics. One study conducted in Italy also highlighted this association.^3^ Patients who develop coronary artery disease and are the candidate of coronary artery bypass graft (CABG) surgery, are likely to have underlying silent carotid artery disease, especially those who are diabetics.^9^ Two major randomized clinical trials showed a potential advantage of carotid endarterectomy in those with severe carotid stenosis of 70-99% in diminishing long term risk of ipsilateral stroke in symptomatic patients.^10^,^11^ Therefore, precise measurement of stenosis is important in deciding patients requiring surgery. As atherosclerosis evolve silently over decades since childhood before clinical CVD, noninvasive imaging modalities like Doppler ultrasound is a widely used tool for the evaluation of arterial wall remodeling, CAS, and carotid intima media thickness. Uncontrolled Diabetes Mellitus (DM), hyperlipidemia, hypertension, increasing age, obesity and hyper-homocystinemia are associated with carotid atherosclerosis.^12^,^13^

As per author’s knowledge, no research work on evaluating burden of silent or subclinical CAS in diabetics and associated factors like age, sex, smoking, duration, and treatment of diabetes etc. have been evaluated in this part of the world. The objective of this study was to determine frequency of silent carotid artery stenosis in diabetic patients. By early detecting silent carotid artery stenosis, asymptomatic diabetic patients can be closely followed up for the worsening of stenosis and evaluated for the need of aggressive medical management or early surgical intervention for the prevention of stroke. In Future this data might help in formulating the guidelines for the timing of intervention for the carotid artery disease with the intension of prevention of stroke among diabetics.

## METHODS

This cross-sectional analytical study was carried out in Medical Unit-II, Civil Hospital Karachi from July 2019 to December 2019 for a duration of six months. The study has IRB approval from DUHS (Ref: IRB-1311/DUHS/Approval/2019/, dated: 24 June, 2019). All patients of either gender aged between 25 to 75 years, with diabetes of both Type-I and Type-II with duration of more than two years were included as macrovascular complications appear late. All those Diabetic patients with the history of cerebro-vascular event or TIA, previous carotid surgery, previous cervical radiotherapy, neck mass (goiter or enlarged cervical lymph node), gross cervical spine deformity (kyphosis, scoliosis or both), causing difficulty in performing the ultra sound doppler were excluded. Carotid artery stenosis was measured by carotid doppler ultrasound of right and left common, internal and external carotid arteries, as mentioned below in detail, by a trained radiologist having an experience of > 5 years, and the reporting was done by the same. Stenosis of ≥ 50% was considered as significant silent CAS.

Carotid Doppler was done by trained radiologist. Duplex measurements of peak systolic velocity (PSV) of the internal carotid artery (ICA) was recorded and the ratio of these velocities in the internal and common carotid arteries (ICA: CCA) was calculated. The criteria determined for detection of 50% or greater stenosis is as follows:

- peak systolic velocity of the internal carotid artery greater than 125 cm/s and
- ratio of peak systolic velocity of the internal carotid artery to peak systolic velocity of the common carotid artery greater than two.

Information regarding other factors was obtained using an interview based self-administered questionnaire. Brief history was taken for age, gender, the duration of DM, compliance to diabetic treatment was noted on history. Information was also sought for home blood glucose monitoring. Smoking status was recorded as current smoker, ex-smoker and never smoker, pack year of smoking was also noted for smokers and ex-smokers. Sampling was done using non-probability, purposive sampling. Taking confidence level (1-α) of 95%, anticipated population (P) of 19.2%^9^ and absolute precision (d) of 0.06, the sample size was calculated as 166 according to following formula.


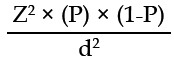


Where Z = Z value (e.g. 1.96 for 95% confidence interval level), P = prevalence of disease, d+ absolute precision of 6% (0.06).

Data was collected and analyzed in the Statistical Package for Social Sciences (SPSS) version 25. Mean ±SD was calculated for age and duration of treatment. Percentages and frequencies were reported for categorical variables like gender, carotid artery stenosis, duration of diabetes and type of treatment, i.e., on oral hypoglycemic or insulin. χ^2^ test was applied to check the independence. A *p*-value of ≤0.05 was taken as significant.

## RESULTS

A total of 166 diabetic patients were included in this study out of which 99 (59.64%) were male and 67 (40.36%) female. Male to female ratio was 1.5:1. The mean age ±SD of the patients was 54.8 ±7.96 years. In this study, there were 27 (22.29%) patients were smoker and all were male. Out of 166 type-II diabetic patients, 59 (35.54%) were treated with insulin and 107 (64.46%) were treated with oral hypoglycemic. Mean duration of diabetes was 8.23 ±0.92 years.

In our study there were 34 (20.5%) hypertensive, 19 (11.4%) were having dyslipidemia while 4(2.4%) had evidence of peripheral vascular disease. All hypertensive were taking anti-hypertensive drugs and their blood pressure was within normal range at the time of induction into study.

Frequency of carotid artery stenosis (CAS) in diabetic patients was observed in 28.92% (48/166) cases. Frequency of carotid artery stenosis was also significantly associated with duration of diabetes (*p =* 0.036), smokers than nonsmokers (45.9% vs. 24% *p* <0.001) and age more than 60 years (*p* <0.001). Whereas there was no significance difference with gender (31.3% in male and 25.4%, *p* = 0.408) and type of treatment for DM (*p =* 0.98), detail presented in Table-I.

**Table-I:** Frequency and factors of Silent carotid artery stenosis in Diabetics.

Variable (n)	Carotid Artery Stenosis		P value	Chi-Square (χ^2^)

	Yes (n=48) 28.91%	No (n=118) 71.9%		
***Age in years***				
<50 (68)	n=14 (20.6%)	n=54 (79.4%)	0.001*	14.31
51-60 (58)	n=13 (22.4%)	n=45 (77.6%)		
>60 (40)	n=21 (52.5%)	n=19(47.5%)		
***Gender***				
Male (99)	31(31.3%)	68(68.7%)	0.408	0.68
Female (67)	17(25.4%)	50(74.6%)		
***Duration of Diabetes in Years***				
≥ 2 (53)	13(24.5%)	40(75.5%)	0.036*	6.63
3-10 (70)	16(22.9%)	54(77.1%)		
>10 (43)	19(44.2%)	24(55.8%)		
***Type of treatment***				
Oral hypoglycemic (107)	31(29%)	76(71%)	0.98	0.0005
Insulin (59)	17(28.8%)	42(71.2%)		
***Smoking Habit***				
Smoker (37)	17(45.9%)	20(54.1%)	0.010*	6.718
Non-smoker (129)	31(24%)	98(76%)		

* Significance level P ≤0.05, n= number of participants.

## DISCUSSION

We found that around one third of diabetic patients have silent carotid artery stenosis, and is specially associated with increasing age, smoking history, and duration of DM. Our findings are consistent with the findings of Chen et al., who reported significant (≥ 50%) stenosis of one or more of the extra cranial cerebral arteries in 21% of patients (32 out of 153 patients) who presented with angiographically documented coronary artery disease, and is associated with Diabetes mellitus, hypertension and peripheral vascular disease.^14^,^15^ Similarly Clarke R et al, who enrolled 24,822 Chinese adults from the China Kadoorie Biobank (CKB) and 2579 Europeans from the UK, reported about one-third of Chinese adults had carotid plaques and the rate of carotid atherosclerosis was increased 10 folds with advancing age (6% at 40-49 to 63% at 70-89 years) in the Chinese compared with the European population, and is more prevalent in smokers than in non-smokers (36% vs 28%) and with higher Systolic blood pressure (SPB) (SBP ≥160 mmHg compared with SBP <120 mmHg; 44% vs 22%) in the CKB study.^16^ Boulos NM et al. in their study also concluded that increasing age is associated with carotid artery stenosis,^17^ which is consistent with our study, because in our study 59% of patients were more than 50-70 years of age. The carotid artery can be considered as a model to reflect conditions common to all arteries.

In our study there was no significant gender difference among those diabetic patient who has CAS, which is in contrast with study of Catalan M et al., who reported high prevalence of preclinical carotid atherosclerosis in women with new-onset T2DM subjects.^18^ This difference may be because of regional or ethnicity origin. One study was conducted in department of Diagnostic radiology in PNS Shifa Karachi on 131 ischemic stroke patients, highlighted the presence of carotid artery stenosis in 56% of patients as measured by Doppler ultrasound.^19^ Another similar study was carried out in Civil Hospital Karachi on one hundred patients with acute ischemic stroke, conclude 39 % of patients found to have CAS where advanced age, male gender, hypertension smoking, ischemic heart disease and hyperlipidemia were the main associated factors.^20^ In contrast to our study these two studies selected patients who already have developed acute ischemic stroke, whereas in our study we enrolled those high risk patients with duration of diabetes of more than two years for the presence of silent carotid artery stenosis with the intent of prevention of stroke. In a recent study old age and duration of diabetes of more than 17.9 years were shown to be significant risk factors for developing silent carotid artery stenosis.^21^

In our study frequency of CAS was 28.9%, which is in contrast with a study conducted in Iran by Tarzamni et al. who reported a very low prevalence of significant (>50%) carotid artery stenosis only in 1.8% (5 out of 271) patients and critical (>70%) stenosis only in 1.1% (3 out of 271) patients.^22^ This difference may be because in his study all patients were not diabetic (reported 28% diabetic patients in their study). Tarzamni et al. included patients who had coronary artery disease and undergoing CABG surgery, whereas in our study we included all diabetic patients with duration of more than two years.

Strengths of our study is that we assessed frequencies and factors associated with silent CAS. There is limited data available in Pakistan in high risk population with the intent of stroke prevention. The tool which was used for assessing CAS was Doppler ultrasound which was a reliable tool.

### Limitation of the study

Since it is cross-sectional study design, no temporal association can be made. Sample size was small. Limited modifiable factors were assessed.

## CONCLUSIONS

A substantial number of diabetic patients have silent carotid artery stenosis. Increasing age, smoking history and duration of DM are strongly associated. Given the high prevalence of carotid stenosis in diabetic patients, this diagnostic approach could be more appropriate to the actual clinical decision-making process.

### Authors’ Contributions:

**FSA:** Designed and conceived the study, wrote manuscript. She is also responsible and accountable for the accuracy or integrity of the work.

**NB:** Collected data, literature search and initial draft write up of manuscript.

**BFZ:** Did statistical analysis and final approval of study.

**SB:** Collected data, literature search and initial draft write up of manuscript.
